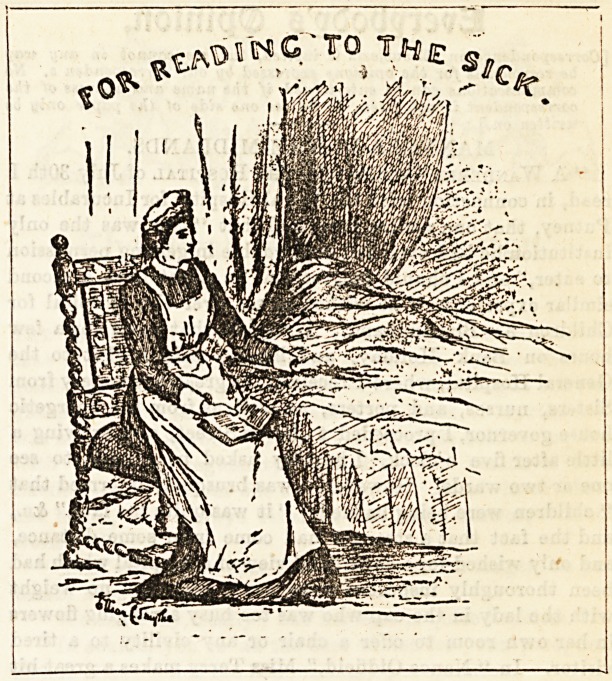# The Hospital Nursing Supplement

**Published:** 1892-08-06

**Authors:** 


### "The Hospital" Nursing Mirror.

Being the Extra Nursing Supplement of "The Hospital" Newspaper.

Contributions for this Supplement should be addressed to the Editor, The Hospital, 140, Strand, London, W.C., and should have the word "Nursing" plainly written in left-hand top corner of the envelope.

### En Passant.

GRAVESEND NURSES.—During the past year the nurses for paying patients of this hospital have nursed in 46 houses, whilst the district nurses have paid 1,521 visits to poor patients in their own homes, and the Nursing Institution generally is progressing. The certificate is given at three years at this hospital, and we hear that bronze badges will be given as well shortly, while an extra two years' work at the hospital will entitle the nurses to a silver badge.

ST. OLAVE'S DISTRICT NURSING ASSOCIATION.—The work of this association will be one year old in October, and it has made an exceedingly good start in a very short time. Miss Watson, the original Superintendent, who came from the Dublin Nursing Association, began with two nurses, but it will be remembered that in the terrible epidemic of influenza in the new year Miss Watson was taken ill and died, to the grief of all connected with the work she had started. Miss Thomas and three nurses are now doing the rapidly-increasing nursing. The nurses' home is 23, St. James's Road, Bermondsey, and all who are interested, from a business point of view or otherwise in this neighbourhood, will do well to send any offering to this good work, which in barely a year's time has so thoroughly justified its existence.

CRIEFF SICK NURSE ASSOCIATION.—The annual general meeting of the Crieff Sick Nurse Association was held under the presidency of Mr. D. Keith Murray. Miss Augusta Murray, Secretary, submitted her annual report, which showed that Mrs. M'Queen, the nurse, had attended 107 families in sickness. She had made an average of ten visits per day, the sum total being 3,969. Nineteen deaths had occurred among the patients. Mr. A. W. Logan, Hon. Treasurer, also submitted his report, showing that the total income for the year was £50 5s. 11d., and the expenditure £63 13s. 5d. There was a credit balance of £4 3s. 2d. An Acting Committee was also appointed. The meeting closed with a vote of thanks to Miss Murray for her services in connection with the association, and to the Chairman for presiding.

WESTON-SUPER-MARE.—The Children's Surgical and Medical Convalescent Home issues its third report; during 1891 one hundred and fifty children were patients, and there were many more applications than could be received. Three more cots have been given during the year all bearing flower titles. We are sorry to find the balance-sheet shows a deficit of £13 13s., but it is not a very startling sum, and it will be easily met out of the year's expenses if more subscribers and friends will come forward. Admission to the Home is at the rate of 6s. per week for children from Clifton and Bristol, 7s. a week from other places, and six weeks is the longest stay that can be made except under exceptional circumstances. The limit of age is eight for boys and twelve for girls. Subscribers of one guinea can place a child in the Home at the reduced fee of 3s. Mr. C. C. Hutchinson, 16, The Avenue, Clifton, or Miss Anderson, the Matron at the Home, will gladly receive the smallest contributions or give any information. If anybody has a spinal carriage to dispose of, perhaps they will remember that this little Home is badly in need of one.

THE Q.V.J.I.N.—In announcing the resignation of Miss Mansell, Inspector of Nurses, at the last meeting of the Council, and the appointment of Miss Peter, the Superintendent at Edinburgh, the following resolution was proposed by the Duke of Westminster, and seconded by Sir James Paget: "That the cordial thanks of the institute be given to Miss Mansell for all the efficient and painstaking work she has done in various ways in connection with the institute since its foundation." Miss Peter will be the third Inspector of Queen's Nurses, Miss Rosalind Paget having been the first to fill the post.

NURSING OF OUTDOOR PAUPERS.—Major Middleton introduced a motion at the fortnightly meeting of the East Preston Board of Guardians that in each relief district of the Union a woman or women should be retained at a small weekly salary, who would be available to attend sick outdoor paupers at the discretion of the relieving officers. The cost, it was urged, would not be great, and the motion was passed. We shall be interested to hear how the newly-appointed Committee decides to work the scheme, and we can only hope it will employ trained nurses or employ the district nurses to see after their cases at a certain cost per case. We shall be very glad if any of our readers will tell us of any Board of Guardians who have to their knowledge made use of the permission of the Local Government Board to employ nurses for their outdoor relief sick cases.

SHORT ITEMS.—At the London Obstetrical Society's examination on the 20th inst., the six pupils sent up from the Manchester Maturnity Hospital were successful in obtaining the society's diploma. This result reflects great credit on the theoretical and practical teaching given by the resident midwives.—A Belfast physician is contributing popular articles on practical nursing to the *Belfast Witness*.—The parish nurse at Witney is giving a series of lectures to members of the G.F.S. on "What to do Before a Doctor Arrives." Her audience is numerous, and the lectures are a complete success.—The third annual friendly societies' demonstration in aid of the funds of the Hampstead Nursing Institution was terminated by a service at the Church of the Good Shepherd.—The *Southampton Observer* has had some good articles, urging the inhabitants to come forward and support a branch of the Q.V.J.T.N.

WELL DONE ABERDEEN!—We read that "the Faculties of Arts and Divinity, and the Science Committee unanimously reported in favour of the admission of women to Aberdeen University," and this just decision is expressed with a graceful cordiality which is especially remarkable as coming from our courteous Scotch cousins. It is pleasant to all who are interested in women's work to find this Aberdeen decision has been arrived at simultaneously with the B.M. Association's Extraordinary Meeting, at which the admission of women was carried by a vote of three hundred against only three or four objectors. Surely this increased liberal-mindedness on the part of their medical brothers will have a distinctly beneficial effect on the race of young lady students, who are yearly increasing in numbers, and, we must hope, in wisdom and womanliness, neither of which should certainly ever be forgotten in the race for fame.

### The Management of Consumptive Patients.

### I.—THE INFECTIOUSNESS OF PHTHISIS.

It is often said, and with some apparent show of truth, that the most uninteresting patient from a nursing point of view, is one with consumption. Again and again has it been urged that in this disease there is little or no scope for the employment of nursing skill. Frequently has it been affirmed, sometimes by high authorities, that for the nursing of consumptives, no great degree of training is required; that, given a person with ordinary intelligence and common sense, as much may be done by her for the consumptive, as by the most completely trained possessor of certificates from the schools. In hospitals devoted to diseases of the chest, one constantly hears the complaint, that after a hard day of trying work there is nothing to show for the labour and trouble bestowed. All this at first sight appears true enough. If we take cases of advanced consumption, here the most assiduous attention will, at best, only eke out a miserable existence, and the nurse has ever before her the picture of a hopeless chronic case gradually progressing to a fatal termination; or if we turn to the early stages of the disease here again the facilities offered for good and efficient nursing are few; the patient is rarely confined to his bed, he is regarded more as a convalescent than a man with actual disease, and beyond the enforcing of rules of discipline, and the performance of a few routine duties, the nurse's services are not brought into requisition. And yet the nurse possesses an immense power, which, if properly used, would become a potent factor in checking the spread of consumption, an influence which could not fail to benefit the patients under her care. What there is in this disease which makes the aid of a properly trained nurse so all-important, and how this skilled aid can best be put into operation, we must now enquire.

Consumption in one or other of its forms is reputed to be answerable for about one-seventh of all the deaths taking place every year. Between the ages of 15 and 45 it kills one-third of those who die, and between the ages of 15 and 35 nearly one-half. In 1882 Professor Koch, of Berlin, demonstrated the presence of a microbe—the bacillus tuberculosis—in the expectoration of consumptive patients. By a series of experiments and observations, which have since been multiplied by others, he established these facts: (1) That the tubercle bacillus is always present in tubercular disease; (2) that these bacilli, when injected into an animal, cause tubercular disease. There is now almost overwhelming evidence to show that the specific cause of consumption, or tubercular disease of the lungs, is this bacillus, a microbe which by appropriate methods can be found in large numbers in the lungs and expectoration of patients suffering from the disease. Immense quantities of this germ are expectorated by a consumptive in the twenty-four hours; some say millions. Let us, in imagination, follow a person with consumption in the every-day walks of life, and determine the destination of these microbes. If a man has consumption he will most assuredly cough; and unless he has been instructed, he will expectorate his germ-laden expectoration, if a poor man, on the floor or in the street; if a rich man, into his handkerchief. In either case the result will be the same; so long as the expectoration is moist it is practically harmless, but whether it be deposited on the floor, where many tread, or in the handkerchief, it soon becomes dry and converted into dust, the particles quickly become disintegrated, the bacilli broken up into spores, and perhaps millions are so liberated, and carried hither and thither by the air. A few years ago Cornet, a German observer, found evidence of these microbes in the dust on the floors, walls, and furniture of rooms in which patients with consumption had been confined, and who had been known to have either expectorated on the floor or in the handkerchief. He further demonstrated the virulent properties of this dust by injecting it into animals, many of those so treated died of tuberculosis. Again, the products of these bacilli may be found on knives and forks and cups and glasses, clothing and linen, in short on anything which has been in contact with a consumptive who has failed to take proper precautions to disinfect and efficiently dispose of his expectoration. Granted then that bacilli gain access to the air from expectorated matters, it follows they must be inhaled in considerable numbers by human beings. It must not, however, be supposed that all persons inhaling microbes into their lungs necessarily become victims of consumption any more than that all persons exposed to the infection of scarlet fever necessarily take the disease. The tubercle bacilli require a certain soil suitable for their growth. If strong, healthy individuals inhale them not much harm is likely to result; but suppose they are inspired into the lungs by a person with a family history of consumption, in other words, by a person strongly predisposed to take the disease, in nine cases out of ten that person becomes consumptive. Or suppose the bacilli gain entrance to the lung of a man whose chest is malformed, or whose work compels him to be in a close and dusty atmosphere, in a cramped and stooping posture, or who, from illness or insufficient food is reduced, and so less able to withstand the onset of the disease; he also stands a fair chance of becoming a victim. This being true, how dangerous the consumptive who takes no trouble to make his expectoration harmless, and how great the responsibility attaching to those looking after him, if they do not faithfully advise him as to his mode of life. It is of commonest occurrence to see consumptives expectorating with impunity in railway carriages and tram cars, on the stones of the streets, and in places of amusement, a peril to all their fellow creatures; but we cannot in justice blame them. They have never been told of the danger. Now all these points, the widespead presence of a specific organism, which is the cause of consumption, the mode of its diffusion through the air, and the results of experiment, prove the disease under consideration to be communicable, to be infective, and consumptives, unless they take proper precautions, must be regarded as dangerous to mankind, indeed, as quite capable of spreading the disease of which they themselves are the victims. They are not, like other infectious individuals, closely confined to a room or a hospital, where they can with safety stay a few weeks until all danger of infection is passed. The consumptives have a disease of years; during that time they have social intercourse with their friends, and enjoy life to no small extent; they consequently have abundant opportunities for spreading their disease broadcast. The belief that because his chest is affected he is thereby more susceptible of cold, leads the consumptive to take care that the room in which he lives, or the workshop in which he works, shall have its windows always closed; he lives with his friends in a close, warm, and stuffy, atmosphere; his supply of oxygen is small, the currents of air in the room few, and the conditions necessary for the bacillus to flourish and gain access to the lungs of those living with him most favourable. It may be conceded that there is little to be done in the actual nursing of consumptive patients, but next to the doctor there is no one who may do so much as the nurse to warn the consumptive of the danger he is to those around him, and who may so readily teach him how to become harmless. Here, in the nurse's work among consumptives, is a golden opportunity for the dissemination of a knowledge which will do much towards diminishing a terrible disease.

### Experiences of Lowestoft Hospital.

INDIA.

Most of the nursing world have been interested lately in Lowestoft Hospital; even scraps of news about it are wafted out here. I know that little hospital well; it is sacred to me, for many pleasant memories; to any Matron less impetuous than myself and endowed with tact it might be an ideal home for any lady—there in the hearts of the fishermen and their wives another Sister Dora might arise. Win the hearts of the Lowestoft people and all else comes. They are called stingy, and they are sometimes much abused, but I found them generous, grateful, and affectionate.

Lowestoft Hospital some years ago was much in debt, but some ladies exerted themselves and worked hard for it, and the hospital was still able to carry on its noble and scientific work. When I went there was great need for retrenchment still. The hospital was under-nursed, the wards were always full, and nearly all were acute cases, and the difficulty of want of sufficient beds often arose. I worked along time with one Sister (a charge nurse), two probationers, and a night nurse. The working was primitive. The accident ward was downstairs, a probationer of three months' standing looked after the men's ward (10 beds) upstairs, and in the accident ward the man who was least seriously ill had a hand-bell beside him, which he rang when either he or any other of the patients required a nurse's attention. There was a child, about two and a half years' old, with a dislocated hip in this ward also, and not a single man could get out of bed. One patient had fallen 40 feet from a mast, having fractured his skull, pelvis, and thigh; another had severe compound comminuted fractures of both ankles.

The Sister and the senior probationer attended to the female ward and the special ward, in which were performed a number of abdominal operations, such as o-ophorectomy, ovariotomy, &c—colotomies and herniotomies being nursed in the general wards. A great number of scientific operations were done in that little hospital with most successful results; the amputations did well, and a few months before I left excision of the knee joint was performed successfully on a woman between 40 and 50, and the bones had united when I left. The cases there were nearly all acute; patients who were operated on in the out-patients' room and afterwards treated as out-patients would have been welcomed as interesting cases in many more famous and illustrious hospitals. Nearly the whole time I was there the work was heavy, and I had at last to ask for more help; it was granted, the old Sister resigned, and I started with two ladies, London trained and of both London and provincial experience, as Sisters or charge nurses. They were surprised at the cases and the operations that were done; some they had never seen.

They found that in emergency they had to set simple fractures, reduce dislocations, stop hæmorrhages, and render first aid to the injured in a way that they had never experienced in their former careers. I never saw nurses work as those Lowestoft nurses did; the probationers were up at six every morning; they had tables, &c., to scrub, and do work that is not expected of many more experienced nurses in larger hospitals. They had an hour off duty one day, and two hours, inclusive of tea, the next. But what compensated for all was one half-holiday a week, and they deserved it. The out-patients took up a considerable time, dressing took always one hour, and occasionally two, every morning; these patients included the casuals, men with accidents who had an hour or two sometimes to spare, in which they could be patched up and returned to sea. There was no house-surgeon, and the honoraries worked hard, they were young and enthusiastic.

In my mind that hospital cannot be properly worked without two charge nurses—call them sisters, if you like—a senior probationer for the accident ward and casualty room, under the immediate supervision of the Matron; a night nurse, who might take it in turn with the accident nurse; and two day probationers upstairs, to learn under and help the charge nurses.

I had to economise to make ends meet. At first I was opposed, but when nurses and patients found that they were as well fed under the new arrangement, and that economy only meant no waste, everyone fell in and assisted me. Women patients made and mended for me; fishermen sent me fish; grateful patients left small contributions for the Hospital Fund; one of the Committee supplied me with firewood free; a patient re-seated and mended all the cane-bottomed chairs for me, and so on. When I left, the hospital account (through the kindness of many Lowestoft friends) showed a balance of £400 to the good.

After they had confidence in me, I found I had my own way in all matters in which it was right I should have it; and if I was interfered with a little, I had only to speak and it was all right. The Committee backed me up, and the doctors were most helpful and the patients most grateful.

The night before I left England my letters were sent on to Portsmouth. Among them were kind ones from some of the medical staff, and from some of the Committee, all expressing good-will and good wishes, and, last of all, one I shall always keep—one breathing of affection from the nurses and servants signed by them all, and conveying good messages from the patients; and I carried away in these letters the assurance that on my outward-bound voyage my safety would be prayed for in the churches and chapels of Lowestoft.

I am sorry to think that Mr. Morse should have cause to think that ladies should be deterred from applying for a post for which eighty applied two years ago. It might, and will, be yet a kind and comfortable home for some Matron. I shall, away in India, consider her to be envied in the comfort of her English home, beloved by the sea-reared Lowestoft people.

E. A. W.

### Wardmaids.

It was accounted a lucky day, in many hospital wards, when a regular servant permanently replaced the casual scrubber who came (or did not come) when required.

From that date the cleaning of bathrooms, grates, and greasy tins; the polishing of brasswork, the scrubbing of lockers, the washing of bandages, and many other details, have ceased to be amongst the duties of either nurse or probationer, and they are probably done as well, and certainly more suitably, by a different class of worker.

Certain fixed rules are drawn out for her guidance by the matron, and these are supplemented by the ward sister, and a copy of the whole usually hangs in a conspicuous place in the kitchen or scullery. After a short probation is satisfactorily passed, the woman is supplied with uniform which has to be worn during all the working hours. It generally consists of strong and serviceable linen or print dresses, with coarse aprons to wear during the rougher labours of the day, and finer ones for use at other times. Caps vary very much in fashion, and the best are those that fit on most firmly, covering a good part of the hair.

In some hospitals there is sufficient space to allow of the wardmaids living entirely on the premises, which is far the best plan, as they are under similar supervision to that which is exercised in an orderly private family, and, therefore, a better class of girl is always available to take these situations. Respectable parents naturally hesitate to allow their daughters to take service under circumstances which necessitate walks at a very early, and also at a comparatively late, hour through London streets, never the safest of thoroughfares for young persons.

When the hospital accommodation does not permit of housing these workers, a certain class of women at once benefits—wives whose earnings are needed for the support of a delicate or lazy husband; and widows, who are thus enabled to keep their fatherless children fed and sheltered. It is a very hard life for these poor souls whose work is literally never ended. Their labours in the hospital wards and lobbies are by no means the most difficult or ungrateful of their tasks. All honour be to those wearied and worn wives and mothers who preserve their integrity and sobriety in the midst of legions of temptations. That portion of the great human family which has never known cold nor hunger will hardly mete out much sympathy to those who fall into those low vices which no halo of sentiment surrounds.

The wardmaid who has had a short night of disturbed slumber in a crowded, unventilated room, awakens at last, in alarm, to find that she has barely time to dress and scurry to the hospital, escaping the odium which a lapse into unpunctually brings, and which, if repeated, risks the loss of the, to her, precious situation. She feels "upset," as she would herself phrase it; exhausted by the bad air she has slept in, probably; and by the want of any food before starting out, and in such circumstances how can we, who breakfast comfortably every day, judge of the extent to which the poor creature is tempted when an older and more hardened companion offers for her refreshment "a taste" of the gin which has composed her own morning drink! If the invitation is declined she is sneered at for being "too good for the likes of us to company with," and worse still, if she accepts, she must, by an unwritten but irrevocable code, "stand treat" herself, next time, and so easily and rapidly join herself to the terribly large contingent of dram drinkers.

However, there always have been and always will be brave and honest women who defy the wrong and cleave to the right, through evil report and good report, and many is the wardmaid whose history might well be written in letters of gold. Some of them keep their places for years, and hold to their old ward though sisters and nurses come and go, and changes take place in every department. Some stick to it because they are fit for nothing else, but others remain because they like no other work so well.

They are a curious race; often holding shrewd opinions of their "superiors" which would cause a rude shock to be experienced if they were uttered aloud.

When a woman has been a long time in one ward she has frequently a very overweening idea of her own importance, and she usually talks of "*Me* and Sister" liking things done in certain ways, &c.

One very worthy worker was much annoyed at that trying moment when the annual cleaning was at hand to find two strange nurses were amongst the ward staff, and uttered her sentiments to another wardmaid thus, "I call it too bad to send us fresh nurses this week. I shan't have a single one on the floor as know where any thing's kept." Her indignation took such a strictly personal form, that it was difficult to remember, for the moment, that she was herself neither responsible for nurses, nor cleaning! All new probationers are looked upon with distrust and criticism by old-established wardmaids, and it rests with the Sister to see that this feeling is kept within bounds. Naturally it is not fair for probationers to have to submit to any rudeness or dictation from that quarter, but, on the other hand, the young nurse would do well to realise that it lies within her powers, by neatness and method, to avoid adding unnecessarily to the labours of a person who has plenty to do. A working woman always respects good management and orderly ways, and she is usually, though not always, obliging to those whom she defines as "not knowing much, but willing to learn." Yes, "willing to learn" expresses a great deal, and especially when it is furthermore defined as "anxious to be taught" we may count on success attending that wish, at any rate. So while condemning the wardmaid who, by her ill-temper and disobligingness, adds greatly to the trials which fall to the lot of each new comer, we may speak with sincere cordiality of the honest woman who not only does her own duty faithfully, but helps other people to do theirs!

### Our American Sisters.

*(Concluded.)*

Our insular pride has often been gratified by the compliments paid by strangers to our luxuriant London trees, appearing as they do in such unexpected nooks and corners, in dull city streets, as well as in our beautiful parks. No one has ever appeared more fully alive to our rich "greenery" than two American ladies recently our guests. They went so far as to accuse us of not being properly alive to our blessings, and of not thoroughly valuing the luxury of having grass that we may "even walk on," such turf as adorns the parks of New York being far too difficult of cultivation in that dry climate for it to be permitted to encounter the dangers of annihilation by any wandering footprints. "It would not grow again with us if we walked on our grass," said one of our observant visitors. The hot June days which have this summer recalled the days of our youth, gave genuine pleasure, as well as an unfairly favourable impression of our English climate, and we naturally fell into talk on the international subject of conversation—the weather! We learnt that, in spite of the lower temperature registered in New York, people seldom feel the cold there as we do here. All the houses are well warmed throughout, chilly passages and freezing bed-rooms being unknown; and the inhabitants are never exposed, when indoors, to those extremes which prevail in many middle-class English homes. Of course, in hospitals the same practice is applied, a warm atmosphere pervading the whole building.

Next, perhaps, to the grass, our open fires elicited most admiration from our guests; they seemed never tired of picturing the cosy winter evenings, when the glory of the vivid flames is specially to be valued, and the useful, clean, but ugly stove is so poor a substitute.

During their first days in London the constantly passing groups or single figures, in nursing uniforms, commanded much curious attention from our friends. From this we learnt that any such costumes are practically unknown in New York; in fact, they would not be tolerated. We wonder whether this will last, or if another half-dozen years will see them do, as we have done, accept the dresses so generally as to lose count of days when such things were not.

But if the Superintendents of nursing in the New World do not provide nor encourage out-door uniform, they certainly have most liberal theories on the vexed question of "laundress." They have all the nurses' washing done in the laundry, which is under their own administration. This being so, we are not surprised to find that a plain-patterned cap, easily "got-up," is preferred, and the pretty, but somewhat elaborate head-gear which is affected by many of our English Sisters, is unknown over there. Becoming as the caps undoubtedly are, we could reckon on the wearers willingly resigning them for a simpler style, if English hospital officials saw their way to defray their nurses' laundry expenses.

Our ward flowers and the numerous handsome plants, growing well in most institutions, were also much admired, and the more important subject of food was also freely discussed.

The diet kitchens, where the American probationers are systematically taught to cook, not only gruel, but birds, jellies, soups, as well as the many ways in which the familiar egg can be served, appear to us to give a far more satisfactory instruction in the culinary art than the somewhat haphazard way in which "sick cooking" is acquired by our English nurses. The New World schools make it a feature in training, not an extra or chance accomplishment.

The serving of patients' dinners was specially observed at the London Hospital, where, at noon, the great lobbies are a busy scene. The large hot-water dishes, with close-fitting lids, in which the food is placed in the kitchen, make it, by means of the lifts, to reach the wards at a delightfully high temperature. Our friends cordially approved of the method by which the distribution of diets was rapidly accomplished.

It is strange that in so many hospitals, both abroad and at home, such grave blunders are made with regard to the best position for a kitchen from which diets are sent to the patients.

If we remember rightly, the John Hopkins' Hospital, which is held up to us, and rightly so in many points, for our admiration and imitation, has all the cooking for patients, doctors, nurses, and servants carried on in a block of buildings inconveniently placed in a corner of the very extensive grounds. Surely, to convey food through the long corridors, which are necessitated by vast buildings only one storey high, is an arrangement opposed to common sense. In the construction of modern buildings it would be well if the convenience of the staff received a little of the consideration which is lavished upon other matters in both England and America.

Nurses' holidays are, on the whole, not so long over there as the annual "leave" which is given here, and we therefore hope that the fact of the shorter working hours in New York balances the longer working year.

It is pleasant to hear that when nurses are sick or in trouble, they naturally return to the hospital which trained them for the shelter or help which is never denied; and does not this prove to us that neither in America nor England could any system of official outside registration compete with that already in force in all well-managed hospitals? Would any University man value a public register as he does his college honours? Would any wise employer of labour judge by such a bald and insufficient record, when the hospital which made the trained nurse what she is is only too willing to give its official report of her work as a supplement to those other testimonials which she has earned since she left her probationer days behind her? A nurse's life is seldom spent in a corner, and when she is "lost sight of" by her hospital, the fault lies in her own indifference or indolence—it certainly does not rest with those to whom she has failed to report herself.

We hope that many more of our American Sisters will find their way into England, for their visits to our hospitals are thoroughly appreciated by the workers with whom they have so many sympathies in common.

### Appointment.

[It is requested that successful candidates will send a copy of their applications and testimonials, with date of election, to The Editor, The Lodge, Porchester Square, W.]

Lecturer for the Leicester County Council.—Miss M. N. M. Didier d'Amblon has been appointed lecturer on Domestic Hygiene and Sick Nursing for this County Council. Miss d'Amblon was trained at the Royal United Hospital, Bath, for one year in 1888; the next two years she trained and subsequently acted as charge nurse and locum to the Sisters at the Birmingham General Hospital. She then went for a short time as charge nurse at West Ham Hospital, and for the last fifteen months she has been district nurse to St. Heliers, Jersey. Miss d'Amblon is an accepted candidate for Netley, and it is while waiting for a vacancy there that she is filling her time as a C.C. Lecturer. District nursing experience is of the greatest value in such lectures, simple practical nursing being the object in view.

### OUR TREASURES.

Some years since there was a young and lovely woman, tall, fair, and graceful, whose life was spread out before her in all the happiness of wedded love. She spoke of her bright lot to a friend, and began to count up the treasures she possessed and valued most. Her husband and children came first, then health and the talents which adorned her position, then wealth, which could give her every desire of her heart. It was in no boastful spirit that she made this catalogue of her gifts, but she felt with the Psalmist, "In my prosperity I shall never be removed, Thou, Lord, of Thy goodness hast made my hill so strong." But as time wore on all was changed. Year after year she lost most of those things which had been her pride and glory. First her husband, then the greater part of her wealth; her children, one by one, were cut off—an only daughter by sickness, the sons in battle on a foreign shore. Worn down by sorrow, the bloom of her beauty departed, and she who in her youth was the envy of her companions, now lay desolate and afflicted, a prey to a lingering and incurable disease. It may be asked, How did she bear this dreary life? At first, with dull despair, she could not see why she had been so pursued by misfortune, and her heart rose up against Him who, she thought, had wrecked her life. But out of the very depths of her despair came the first glimmer of light. She was frightened at her own awful thoughts, which left neither hope for this world nor the next, so she cried unto the Lord in her trouble, and he delivered her out of her distress. Gradually, and very slowly, the darkness passed away from her soul, the Lord Himself shone upon her with the light of His countenance. Oh! the struggle to grasp the pierced hand held out to her. Oh! the silent but deep murmerings, the repinings to be wrested with before she could take for her treasures the gifts now thrust upon her. By God's help she came off more than conqueror. Slowly, but surely, a great calm fell on her heart and she could recognise the Giver who was now pouring His blessings sevenfold into her bosom. Once again she counted her treasures; how changed they were! In long months of anguish pain had forged for her the bright and stainless shield of "Endurance"; she had found in her wanderings in the misty caverns of doubt the "peerless jewel, Faith"; her intellect, quickened and refined by sorrow, broke forth into poetry, and she "learnt in sorrow what she taught in song." The strife about her property, which racked her spirit, left the flower of "Patience" blooming on her breast, and the sufferings she so much dreaded "laid the fair child 'Pity' smiling in her arms." She realised the promise of "beauty for ashes," and rose on stepping stones of her dead self to higher things. She waited in patience for the summons to join her store of loved ones in Paradise, and with them to enter into the joy of her Lord.

### Everybody's Opinion.

*[Correspondence on all subjects is invited, but we cannot in any way be responsible for the opinions expressed by our corresponden s. No communications can be entertained if the name and address of the correspondent is not given, or unless one side of the paper only be written on.]*

### MANNERS IN THE MIDLANDS.

"A Wanderer" writes: In The Hospital of July 30th I read, in connection with the Royal Hospital for Incurables at Putney, that one visitor remarked that "this was the only institution he had ever had any trouble in getting permission to enter," and I should suggest that he might have a second similar experience if he wished to see over the Hospital for Children at Birmingham. I was in that town for a few hours on Bank Holiday, and having paid a visit to the General Hospital, where I received the greatest courtesy from Sisters, nurses, and porters, as well as from the energetic house governor, I proceeded to Broad Street, and arriving a little after five o'clock, I humbly asked permission to see one or two wards. However, I was brusquely informed that "children were being bathed," "it was quite too late," &c., and the fact that a stranger had come from some distance, and only wished for a superficial view of a hospital which had been thoroughly inspected some years ago, had no weight with the lady in the cap who was too busy arranging flowers in her own room to offer a chair or any civility to a tired visitor. In "Nance Oldfield," Miss Terry makes a great hit when she asks her surly visitor if he won't sit down, as a pretty plain hint that it is his place to offer a chair to herself, and really it seems as if a few such practical lessons in manners would be well bestowed elsewhere. As a contrast to this inhospitable reception, I am reminded of a hot day last summer, when two ladies, excusably confused between the three infirmaries which have Highgate as their address, were shown into the Matron's room at Dartmouth Park Hill, and discovered, to their dismay, that they had come to the wrong place. But the dismay was short-lived, for their courteous hostess, moved with pity at the fatigue and disappointment of the strangers, insisted on their resting and on refreshing them with tea. Need I add that when they returned to their country home they took with them a very pleasant impression of a courteous Matron?

### HOSPITAL AND ASYLUM NURSES.

"Caritas" writes: Dr. Greene and "Asylum Nurse" are not the only ones who feel that the reviewer of "Asylums of the World" might have moderated his ardour in speaking of the relative training and standing of asylum and hospital nurses. To say that not a ray of the light of enthusiasm has penetrated asylum gloom is not true; to say that "no apostle" of a new order of things has arisen may be nearer the mark. But we do not want an apostle; we who think we know something of asylum life know that improvement is coming both to the character and training of the attendant. It may be a slow process, but it is better that it should come slowly from individual nurses convinced of their own ignorance than in one of the bursts of frantic enthusiasm so common in these days with which followers rush after a new apostle and leave him and his doctrine as quickly as they joined. We may not all put any value on the Medico-Psychological Certificate *per se*, but you recently published a list of those who have shown enterprise and a wish for knowledge in having at any rate gone up and gained it by passing the examination, which is doing excellent work as an incentive to attendant training. I have also noticed the very numerous notices compared to former years, which tell of the classes who have gone in for the ambulance examinations. Perhaps your reviewer can explain what motive these attendants had in joining these voluntary classes if "no ray of light" had penetrated to them? Anybody who knows at all about the subject will concede that it is all very far from ideal yet, and it is also true that it is the few and not the many who have thought at all about how insane patients are nursed; but it is the system at fault, and we must strike at the roof of the matter and convince the Medical Superintendent as to training instead of blaming untrained attendants. But I would like to know about one thing in Dr. Greene's letter which, when we think of the progress at Berrywood, strikes one as justifiably "enthusiastic"; why may we rest assured "that no Medical Superintendent will move in the direction shown by the hospitals?" What direction? I ask in all sincerity, being much interested in the question, will Dr. Greene tell us?

### NURSING AT CARLISLE INFIRMARY.

Miss C. A. Allen, the Matron, writes: Some erroneous statement having appeared in a nursing print of this week as regards the length of time considered necessary for a nurse's training, I should like to enclose the rules always in use at this infirmary, and which have been upheld by me during my matronship subject to the committee's entire approval. It would be far better if facts were ascertained before such grossly incorrect comments were made. My not being a member of the B.N.A. has doubtless been the cause of such a letter being written. In every hospital a probationer can be trained for one year provided she pays a fee, and they have been sent here from other institutions for one year's training only, to fit them for private nursing—this hospital, therefore, proves no exception to the rule; for our own private nursing staff probationers have to train for three years

The following is a copy of the Regulations for Training Probationers at the Cumberland Infirmary, Carlisle:—

Probationers are received on the following conditions:—

(1) Probationers to be bound for 3 years.

Rate of Wages as follows:—

1st year (including month's trial), no payment.

2nd year, £16.

3rd year, £20.

During their third year they shall be available for Private Nursing.

Indoor Uniform consisting of Dresses, Caps, and Aprons, will be provided. They will be expected to find their own out-door Uniform.

A Certificate of Training will be given at the end of the second year.

(2) Special Probationers coming for twelve months' training to pay at the rate of £30 a year, or £1 1s. a week for a shorter time, and to find their own Indoor and Out-door Uniforms.

If satisfactory, a twelve months' Certificate of Training will be given.

(3) All Probationers to pay an Entrance Fee of £1 1s.

If found satisfactory at the end of the month's trial, Probationers will be required to enter into the following agreement:—

Having become practically acquainted with the duties of a Nurse, I agree to serve the above Infirmary in that capacity, and in whatever situation the Matron may place me, for the space of three years, and if desiring to be freed from this engagement before the expiration of the term agreed upon, I undertake to give three months' notice and pay £20 by way of forfeit.

### LONGTON COTTAGE HOSPITAL.

Miss S. Galwey, Dorchester, writes: Having read with interest and no little amusement the paper controversy which has been carried on for the past few weeks relative to the re-appointment of the Matron of Longton Hospital, I venture to make a few remarks on the subject. It is no unusual occurrence to re elect a Matron, and a similar event was enacted at the Dorset County Hospital, Dorsetshire, six months ago, where, within a few weeks of the date fixed for the election, the Matron was asked to re-consider and withdraw her resignation, which she did. I was a candidate, but not a "disappointed one," feeling sure that the re-elected Matron was "the right person in the right place,' and the action of the committee proved that they were of the same opinion.

"Lover of Justice" writes: Reading the remarks concerning Longton Cottage Hospital has reminded me that about six weeks since a charge nurse was advertised for at a London infirmary. A number of nurses applied for the vacant post by appearing on the day of election, as requested in the advertisement. Evidently the vacancy was to be filled by promoting one of the staff, who was seated among the candidates dressed in indoor uniform. Now, Sir, is it not most unfair to bring women (some a long distance) to apply for a post that is to be filled by promotion? As an infirmary nurse, I am most keen on promotion; but it does seem quite inexcusable to treat nurses in this way. Seeing the advertisement for charge nurses again this week, I would like to warn any intending candidates that the vacancy may be already filled.

### "Lady Sanitarists."

The *Daily News* does not often make such blunders as the one which occurred last week in heading a notice of the proceedings at the Parkes Museum as above. The occasion was the distribution of certificates to a few lady students, who had passed an examination which followed some popular lectures on domestic hygiene by Dr. Schofield. Now these are all very well for lady amateurs, and so are the fashionable ambulance classes for those who cannot go deeper into the subjects of nursing or hygiene, but we strongly object to the term of "Lady Sanitarists." There are only two women in England who have passed the regular examination of the Parkes Society, and one of these ladies did so in 1891, and was a favourite lecturer of the N.H.S. The second candidate passed successfully in the spring of the present year, and is a member of the old original Ladies' Sanitary Association in Berners Street, and is one of its most popular lecturers on hygiene and nursing. We trust there will be many followers of these pioneers in a most useful path of knowledge for all mothers and daughters to walk in.

### Where to Go.

The Governors of the People's Palace Technical Schools at Mile End are going to open a collection of pictures at Queen's Hall, on August 15th. The pictures are by the best artists, and admission is free.

### Notes and Queries.

### Queries.

Can anyone kindly inform "Little Jim" what are the component parts of "chloralamide," and if any ill-effects would be likely to ensue from a patient taking it continually for three months (15 to 30 grains)? Is it accumulative?

### Answers.

*Private Nurse.*—Do they teach it at the Cheltenham General Hospital and Dispensary? You had better inquire there. If not, you could certainly take lessons from any certified chemist. Your ultimate aim must be to pass the examination of the Society of Apothecaries—fee two guineas. You would have to make arrangements, of course, as to what terms you were taught on. "Practical Dispensing," by C. J. S. Thompson, would be of great help to you; or, if you can get to a school, ask what handbook they advise.

*E. M. L. Boscastle.*—Apply to Major-General Oliver-Newmarch, C.S.I., Military Secretary. India Office, Whitehall, S.W., for all information as to Lady Roberts' Fund for Nursing Sisters and Officers' Hospital. A written answer has been sent you, and we shall be always very glad to help you; it is no trouble.

*Sister Lucie.*—We are obliged to stick to the rule first come first served, and the correspondence last week came before your note, which we have inserted with pleasure this week. If we did not take things in order we should never be up to date.

*F. W. H. C.*—They give you a two years' curriculum, but you can only become a medical woman through one of the recognised schools in this country—London, Edinburgh, or Dublin.

*Nurse H.*—We are very sorry, but we could not put your question in, or we should be inundated with gratis advertisements. We hear that the Hollond Institution, 1, Tavistock Chambers, Hart Street, Bloomsbury, have vacancies for four months, £2 10s. a month, everything found; it is worth while writing. We know they are looking out for nurses. Did you mean your query to go in the paid advertisements, I wonder? Will you send a post-card if you wish this done.

*R.*—A correspondent recommends rooms at 18, Quarry Street, Guildford, opposite the old Castle Gate, for a nurse's holiday; terms very moderate.

*Nurse M.*—See answer to "Private Nurse."

*Canary Islands.*—We are making inquiries for you, and will let you know as soon as possible.

*Alice.*—Will answer next week.

*Nurse E. G.*—Thanks for note; I put the note in the "Mirror," and am glad it was of use. There is an institution in the place as you know. Someone I asked doubted your success, but I have asked another friend for advice.

### Wants and Workers.

Wanted, a home (in small institution preferred) for an orphan girl (24) requiring firm, kind treatment. Willing to do light house work under supervision, and able to pay 8s. weekly.—Address, with full particulars, *Sister Lucie, Littleborough, Lancashire.*

*Home for an Epileptic.*—A lady writes to ask if anybody can tell her of a home for a young girl who is suffering from epilepsy, with no hope of cure, and now a patient in an infirmary. Will any of our readers tell us if they can help in naming a home for this case?

### The Real Mrs. Skeffington.

*(Concluded from page cxxviii.)*

The days soon came when the village heard, with concern, that Mrs. Skeffington had taken to her bed. After that the gentle, pale sufferer, of whom the village had caught but rare glimpses as one and another passed the gates of Myrtlebank, was never seen again. Widow Wigsby made bold to inquire, with persistent regularity, regarding the health of Mrs. Skeffington, whenever she encountered "Miss" in or about the house, and always received the same answer, "Much weaker!"

"'Tain't in nature that it can go on much longer, do you think, Miss?"

"We can't say, really; but her case is quite hopeless."

"In course, you've 'ad the best advice for your pore, dear sister, Miss?" was the insinuatingly put question.

"Yes, oh, yes, of course! And the doctor, you know, Doctor Skeffington, is in devoted attendance on my sister."

"I make no doubt, miss, of that; and a terrible handy thing to be sure it must be to 'ave a doctor for an 'usband; saves them bills, and should make a death-bed real comfortable, *I* say."

The widow's listener gave her a startled look; then presently observed, with what seemed to be studied calmness, "We have no hopes whatever of Mrs. Skeffington's recovery. We are quite prepared for the worst."

"Deary me! Pore dear! And I shouldn't be surprised if so be as she went off sudden, in the end, Miss. Well, its what we must all come to, and you knows where to find Martha Wigsby, Miss."

"Miss" shivered perceptibly at the gruesome hint, and hastily edged away.

"James," said she, however, that evening, "will it never come? I want to get out of this melancholy hole. I'll die myself, in reality, if you don't take me away."

"You! Well, that would be rather a joke!" The grave doctor raised his black eyebrows whimsically. "Not a bit of it, Julia. Think of the fine times before you, when we get all that pile of money. Shan't we make it fly! Five thousand!"

"Hush—h!" warned Julia.

"Who's to hear me? Cook can't. It was a splendid chance for us to get that deaf and dumb woman, wasn't it?"

"It was," said Julia, with a hard laugh; "you should see the fun when that prying old charwoman tries on a pumping conversation with her. But now, tell me frankly, how long will it be, James?"

"Possibly four weeks," was the slow reply. "Of course, the stimulants and the nourishment are actually keeping life in her. If they were discontinued abruptly, say, there would be a sudden collapse; but"— the dark, beady eyes were rivetted on Julia's face—"if the supply were even cut down to half, the end would be rapidly hastened."

There was a silence in the room, and a long look passed between the speakers, an understanding look; but no more words were said—perhaps no more were necessary.

Shortly after this conversation the village was surprised to learn that "Miss" had run up to London for a few days' shopping. Who in the world was nursing the invalid, was the natural question. The doctor might be seen every fine morning setting off to the distant woods, with his tin specimen case, on botanising expeditions—his special hobby. Surely the deaf and dumb cook was no fitting attendant. After the manner of small communities, people passed censorious judgments, and felt uneasy in their minds, for who was to know what might happen any hour. "Besides," as Widow Wigsby truthfully remarked, "such an incapable old body as that cook couldn't know just when to administer the necessary medicines, and equally necessary nourishment."

A sort of ferment of excitement sprang up; but "Miss" returned, and it subsided for a brief spell, only to break out afresh one sunny morning when it was discovered that the blinds at Myrtlebank were still drawn down. At once a hue and cry was raised for Widow Wigsby, but that individual was not visible, and it began to be whispered about that she had been called up at dawn to go to Myrtlebank. Not until her arrival home again was public curiosity satisfied. Yes, the drawn blinds were significantly true. Mrs. Skeffington was dead! Fortunately, the doctor from the next village, who was hurriedly sent for, had got to Myrtlebank a full hour before the death. The doctor from the next parish! Barrow-cum-Easter stared, and its own medical man, Doctor Clay, looked grim first, and supercilious next. Thus far went the charwoman's information and no farther. A remarkable change came over her, and puzzled people went the length of hinting that the worthy woman had seen a something, that is of the ghostly order, in the house of mourning.

"That's as may be!" was the suggestive, if terse, reply they got. Even the funeral, a rather ostentatious affair as regards show, did not rouse the altered widow out of her oyster-like reserve into any further communications.

After the funeral there was a lull. Myrtlebank became isolated, for Mrs. Wigsby's services were no longer required in the diminished household, so all intercourse was thus cut off. The doctor soon began to be seen on his customary tramps after specimens, accompanied by "Miss," in her new mourning. But communication between the village and the afflicted home had ceased. It is true the parson, in duty bound, called to administer consolation, but he was given to understand, by signs from the dumb domestic, that nobody was at home.

Doctor Masters, from the next parish, drove over and left a card also; indeed, the latter was the only person who had had access to the household. It was from him, therefore, it leaked out that the life of the poor deceased lady was heavily insured—for several thousands, it was believed, in various offices—and doubtless Doctor Masters, who would have the signing of certificates, ought to know. Doctor Clay, who had not got over a certain grudge at not being called in, made it *his* business to write to his brother-in-law, who was in one of the head insurance offices, and the report was proved to be a fact, as Mrs. Clay herself assured Widow Wigsby.

It was after this that the change in the last-named worthy assumed new and startling features. She took to her bed and requested, with a firm mien to be visited by the Squire, who was a J.P., or, failing his worship, any "p'leeceman" would do, she announced. The neighbours tapped their foreheads significantly, and fetched Dr. Clay instead.

"Now, then, Mrs. Wigsby, what's ado with you?"

"Me, sir? Nothin', sir! Its only as I've somethin' on my mind," was the dogged reply.

"Out with it, then, my good woman!"

"Well, sir, it's about that there willin at Myrtlebank——"

"Dr. Skeffington, d'ye mean? How dare——"

"Him a doctor! Ee ain't one, no more an' I am, sir. And that pore dear was no wife of his. She was our Miss Em'ly, if so be as my name's Martha Wigsby, and it's my belief, sir, as them two made away with her!"

Dr. Clay pricked up his ears. Then, regarding his patient, whistled; but for all that, he listened with avidity. Mrs. Wigsby, it appeared, had lived in her native place as "general," to quote herself, with two maiden ladies. Years after one of the sisters died; the remaining one went to live with a person who passed for a doctor. This fact Mrs. Wigsby knew from a married daughter of her own, who kept her up in the local news.

"And I declare to you, sir, solemn as solemn, that corp as was buried four weeks come a Toosday, was our Miss Em'ly!"

"Your proofs!"

"Didn't I know her at once! Besides, there was the marks on Miss Em'ly's arm and wrist where she was bit by a retriever, which it caused her 'ealth to break down. And the little finger of her right hand was took off at the first jint when a girl. Yes, I knowed that corp was our Miss Em'ly at once, and it did give me a turn. But whatever it all means *I* can't guess!"

But Doctor Clay guessed, after a long spell of thinking. And then he set to work. First, he found out that the navy contained no "Doctor Skeffington," past nor present. Neither did the Medical List. So far, so good; Doctor Clay rubbed his hands. The next step was to put the matter into the hands of his brother-in-law, and further investigations were set on foot.

But, meanwhile, the Myrtlebank blinds were again found drawn at noon; this time, quite another kind of departure had taken place. Doctor Skeffington and "Miss" had left—in the night—and the deaf and dumb cook found herself in possession of the house and furniture. Unfortunately, the various insurance offices had each paid up, and the now almost wealthy pair had absconded!

However, the summer was not well over before they were picked out, by skilled detectives, from among the fashionable crowd of a Continental *pension* spending their ill gotten gains recklessly. The discovery of the unique fraud created a great sensation. Barrow-cum-Easter was in every man's mouth, and, in consequence, the village quite took on airs at such notoriety. As for Mrs. Wigsby, that good woman became so exalted in mind, that it was thought to be only friendly to point out how, according to all precedent, pride invariably walked before a fall.

Really, the nine-days-wonder exemplified the old saying that truth is stranger than fiction, for it was found that a clever swindle had been thought out, and carried out as well. The Skeffingtons, man and wife, had tried, in their time, various modes of earning a living, before they advertised for "invalid ladies to reside in a married doctor's home, delightfully situated, and all the rest of it." But they had not been successful; nor were they in obtaining invalid ladies, with one exception, but "our Miss Em'ly's" money was useless to float their latest venture, so a bigger swindle was devised. Mrs. Skeffington's life was insured, and, she being a sound, healthy woman, considerable sums were staked. In due time Myrtlebank was hired and furnished, the Skeffington's, with the sole inmate of their home for invalids, poor Miss Em'ly, all unconscious of the use she was being put to, arrived to await there the termination of her already frail thread of existence. It was given out, by means of Widow Wigsby, that the elder of the ladies was the wife of the doctor, and when death came to set her free from suffering, the neighbouring doctor called in, and was made, by the help of a few misrepresentations, to believe the same fact, in order that he should aid in the deception, by his certificate, to that effect.

Everything would have gone on prosperously, and "our Miss Em'ly" would have slept, for all time, under the headstone that signified she was the wife of Doctor James Skeffington, save for the humble charwoman.

Nemesis thus dogged the heels of the guilty pair, in the good old fashioned way that always brings down the house; and justice to the villains of the piece was promptly dealt out in the shape of transportation for life.

M. B. Manwell.

## Figures and Tables

**Figure f1:**